# Genetic Variants of Gonadotropins and Their Receptors Could Influence Controlled Ovarian Stimulation: IVF Data from a Prospective Multicenter Study

**DOI:** 10.3390/genes14061269

**Published:** 2023-06-15

**Authors:** Carlo Alviggi, Salvatore Longobardi, Enrico Papaleo, Daniele Santi, Simona Alfano, Valeria Stella Vanni, Maria Rosaria Campitiello, Pasquale De Rosa, Ida Strina, Ilpo Huhtaniemi, Juha-Pekka Pursiheimo, Thomas D’Hooghe, Peter Humaidan, Alessandro Conforti

**Affiliations:** 1 Department of Public Health, University of Naples Federico II, Via Sergio Pansini, 80131 Naples, Italy; 2 Merck Serono S.p.A., 00176 Rome, Italy; 3 IRCCS San Raffaele Hospital, 00163 Milan, Italy; 4 Unit of Endocrinology, Department of Biomedical, Metabolic and Neural Sciences, University of Modena and Reggio Emilia, 41121 Modena, Italy; 5 Department of Neuroscience, Reproductive Science and Odontostomatology, University of Naples Federico II, Via Sergio Pansini, 80131 Naples, Italy; 6 Department of Obstetrics and Gynecology and Physiopathology of Human Reproduction, ASL Salerno, 84124 Salerno, Italy; 7 Azienda Ospedaliera Universitaria Federico II di Napoli, 80131 Naples, Italy; 8 Faculty of Medicine, Department of Surgery and Cancer, London W12 0NN, UK; 9 Institute of Biomedicine, University of Turku, 20014 Turku, Finland; 10 Department of Development and Regeneration, KU Leuven, 3000 Leuven, Belgium; 11 Fertility Clinic at Skive Regional Hospital, Faculty of Health, Aarhus University, 8000 Aarhus, Denmark

**Keywords:** ART, IVF, polymorphism, genetic variants, ovarian stimulation, gonadotropin

## Abstract

Background: Specific polymorphisms might influence controlled ovarian stimulation in women undergoing assisted reproductive technologies (ARTs). Data regarding possible interactions of these polymorphisms are still scanty. The aim of this analysis was to evaluate the effect of polymorphisms of gonadotropins and their receptors in women undergoing ART. Methods: A total of 94 normogonadotropic patients from three public ART units were enrolled. Patients underwent a gonadotropin releasing hormone (GnRH) long down-regulation protocol with a starting dose of 150 IU of recombinant follicular stimulating hormone (FSH) daily. Eight polymorphisms were genotyped. Results: A total of 94 women (mean age 30.71 ± 2.61) were recruited. Fewer fertilized and mature oocytes were retrieved in homozygous carriers of luteinizing hormone/choriogonadotropin receptor (LHCGR) 291 (T/T) than in heterozygous C/T carriers (*p* = 0.035 and *p* = 0.05, respectively). In FSH receptor (FSHR) rs6165 and FSHR rs6166 carriers, the ratio between total gonadotropin consumption and number of oocytes retrieved differed significantly among three genotypes (*p* = 0.050), and the ratio was lower in homozygous A/A carriers than in homozygous G/G and heterozygous carriers. Women who co-expressed allele G in FSHR-29 rs1394205 and FSHR rs6166 and allele C LHCGR 291 rs12470652 are characterized by an increased ratio between total FSH dosage and number of oocytes collected after ovarian stimulation (risk ratio: 5.44, CI 95%: 3.18–7.71, *p* < 0.001). Conclusions: Our study demonstrated that specific polymorphisms affect the response to ovarian stimulation. Despite this finding, more robust studies are required to establish the clinical utility of genotype analysis before ovarian stimulation.

## 1. Introduction

Several studies demonstrated that individual gonadotropins and the genotype of their receptors could affect ovarian stimulation (OS) [1,2,3]. Among these receptors, the FSHR polymorphism located in amino acid position 680 of exon 10 (FSHR N680S; rs6166) has been the most widely investigated. In fact, previous studies showed that FSHR A680G G homozygous women need a higher amount of exogenous gonadotropin during OS [4,5,6] and had higher basal FSH levels than women carrying other haplotypes [2,7,8]. In addition, the polymorphism in the 5′ untranslated region of FSHR (rs1394205) seems to exert an influence on ovarian response to exogenous gonadotropin. In detail, FSHR-29 (rs1394205) A homozygous women had fewer oocytes retrieved and a lower clinical pregnancy rate than did GG homozygous women [9,10]. Similarly, fewer mature (MII) oocytes were retrieved in carriers of the FSHR-29 (rs1394205) AA genotype than in carriers of the GG genotype [10,11]. However, a higher live birth rate was reported in FSHR-29 (rs1394205) A homozygous women than in women with other haplotypes in a retrospective analysis of 603 women undergoing ART [12].

An increased exogenous FSH consumption was observed in carriers of the luteinizing B subunit (LH-B) W8R/I15T rs1800447 genetic variant of the luteinizing hormone (LH) β subunit [13] (Alviggi et al., 2013). This polymorphism presents a reduced half-life in vivo in contrast with the wild-type form [14] and is widely expressed in Northern Europe and in Australian aboriginal populations [15].

In another investigation involving 384 women undergoing ART, the pregnancy rate was higher in carriers of the LHCGR 312G polymorphism than in A312 carriers (LHCGR A312G rs2293275) [16]. Furthermore, LHCGR 312 G homozygotes required a higher amount of gonadotropins for follicular recruitment than did A homozygotes. The LHCGR A312G rs2293275 polymorphism was also implicated in polycystic ovarian syndrome development (PCOS), with higher risk of PCOS in AA homozygotes [17]. When combined, gonadotropin receptor polymorphisms could also modulate OS [18]. For example, the pregnancy rate was higher in women homozygous for G than in women homozygous for A in both the FSHR A680G and LHCGR A312G polymorphisms [16]. Furthermore, in a retrospective study, homozygotes of both AA FSHR-29 and AA FSHR A680G had an increased risk of impaired ovarian response after OS [19]. Moreover, carriers of the FSH B subunit (FSHB)-211 GT plus FSHR 2039 AA genotype had significantly lower day 3 FSH levels versus carriers of the FSHB-211 GG/FSHR 2039 GG genotype [20]. However, most of the studies that investigated the role of polymorphisms in OS have some limitations. For instance, most studies were retrospective and were widely heterogeneous in terms of the ART protocols used and patients recruited [4,6,9,10,12]. Moreover, little is known about the interactions, if any, among these genetic variants.

The aim this multicenter prospective study was to assess the effect of multiple genetic variants of gonadotropins and their receptors in women undergoing OS with the GnRH agonist long down-regulation protocol and a fixed FSH starting dose.

## 2. Materials and Methods

### 2.1. Study Population

Caucasian women from three ART centers (University of Naples Federico II, IRCCS San Raffaele Hospital, Milan, and Aarhus University, The Fertility Clinic, Skive Regional Hospital, Denmark) were enrolled in this study. We recruited women with the following characteristics: age from 20–35 years; body mass index (BMI) from 20–27 kg/m^2^; basal FSH ≤ 10 IU/L; indication for ART; presence of both normal ovaries; normal ovarian reserve according to Patient-Oriented Strategies Encompassing IndividualizeD Oocyte Number (POSEIDON) [21,22] (antral follicle count > 5). Women with the following characteristics were excluded from the analysis: abnormal uterine cavity; endocrine, genetic, or systemic inflammatory-immunological disease; presence of PCOS according to the Rotterdam criteria; and diagnosis of endometriosis. Moreover, we excluded women with a history of more than two previous ART procedures that had a good ovarian response or a previous stimulation cycle that had been suspended due to an inadequate ovarian response or in whom < 4 oocytes had been obtained.

### 2.2. Stimulation Protocol

The GnRH-a long down-regulation protocol was adopted using buserelin acetate as follows: 0.5 mg s.c. daily from luteal phase for 12–14 days. Fourteen days later, transvaginal ultrasound and biochemical evaluations were performed to verify the pituitary suppression. Only women with a serum estradiol (E2) level ≤ 40 pg/mL, an endometrial thickness ≤ 5 mm, and arrested follicular development were admitted for controlled OS. Women with delayed pituitary suppression (including subjects who developed ovarian cysts after GnRH-a administration) were not included. A fixed starting dosage of 150 IU of recombinant human FSH (r-hFSH) was adopted for all participants (Gonal-F^®^; Merck Serono S.p.A, Rome, Italy) for at least four days. E2 was measured on day five of stimulation. On that day, the daily dose of gonadotropin was modified when E2 concentration > 180 pg/mL. Only in these cases was a daily dose of r-hFSH of 112.5 IU adopted according to standard clinical practice. Follicular growth was evaluated on day 8 of stimulation by transvaginal ultrasound. Only patients who had at least 6 follicles ranging between 6 and 10 mm in diameter, but no follicle with a mean diameter > 10 mm, received an increased dosage. Specifically, the r-hFSH dose was increased by 150 IU per day, for a cumulative daily dose of 300 IU. Women who had their daily dose of gonadotropin reduced on the fifth day of stimulation and who required another increase on day 8 were excluded from the study. Women who required “coasting” to reduce the risk of OHSS were also excluded. E2 serum levels were measured on days 1, 5, and 8 of stimulation and on the day of administration of human CG (hCG). According to clinical practice, the ovulatory dose of 10,000 IU of hCG or 250 mcg of recombinant hCG was prescribed in women who had three follicles with a mean diameter of at least 17 mm. Oocytes were collected by the transvaginal ultrasound approach 34–36 h after hCG injection. Embryo transfer and luteal phase support were conducted as previously mentioned [13,23].

### 2.3. Polymorphism Analyses

Venous blood samples were clotted and centrifuged at 400× *g* for 10 min. Serum was divided into a maximum of four aliquots and frozen. Pellets were also separated into four aliquots and stocked at −80 °C.

We used an amplicon-based next-generation sequencing assay (TruSeq Custom Amplicon v1.5, Illumina, San Diego, CA, USA) to genotype the following SNPs: (i) FSHR 307 rs6165; (ii) FSHR 680 rs6166; (iii) FSHR-29 rs1394205; (iv) LHCGR intronic rs4073366; (v) LHCGR rs2293275; (vi) FSHB 2623 rs6169; (vii) v-LH rs1800447. The probes for custom panels targeting the LHB, FSHR, LHCGR, and FSHB genes were designed with Design Studio (Illumina, San Diego, CA, USA) and consisted of 44 amplicons with an average size of 250 bp and a cumulative targeted region of 5.7 kb. Polymorphisms were avoided in the primer design. The pooled libraries were paired-end sequenced (2 × 151 bases) with V2 chemistry on a MiSeq instrument. The PhiX control library (5%) was spiked in each run to estimate the sequencing error rate. Polymorphisms were analyzed with VariantStudio (Illumina, San Diego, CA, USA). The HCGR 291 rs12470652 polymorphism was genotyped with bidirectional Sanger sequencing by using a BigDye Terminator v3.1 Cycle Sequencing Kit (Applied Biosystems, Foster City, CA, USA; Eurofins Genomics, Ebersberg, Germany).

### 2.4. Primary and Secondary Endpoints

The primary endpoint was the ratio between cumulative r-hFSH dosage and mean number of oocytes obtained. Secondary endpoints were: E2 levels on the day of hCG, cumulative dosage of r-hFSH, number of preovulatory follicles, number of oocytes retrieved, the number of mature oocytes retrieved (MII oocytes), number of fertilized oocytes, number of embryos transferred, implantation rate, pregnancy rate per cycle, pregnancy rate per transfer, clinical pregnancy rate per started cycle (presence of embryos with heartbeat), clinical pregnancy rate per transfer (presence of embryos with heartbeat).

### 2.5. Statistical Analysis

Genotype distributions of the SNPs evaluated were collected by direct computing, and linkage disequilibrium was evaluated using SNPStat. Hardy–Weinberg equilibrium was measured [24]. The chi-square test was conducted to compare the frequencies of SNPs in the enrolled patients to those of the general population using widely available databases. For each polymorphism evaluated, the codominance model was considered, supposing that the contribution of both alleles was visible in the phenotype.

The Kolmogorov–Smirnov test was carried out to evaluate the distribution of variables. Differences in continuous variables among groups were evaluated with an ANOVA univariate model for normally distributed variables, and the Kruskal–Wallis or Mann–Whitney test for not normally distributed variables. The Dunnet test was used as a post hoc test. Spearman’s rho regression was performed for bivariate correlation. Statistical analysis was carried out using the Statistical Package for the Social Sciences software for Macintosh (SPSS Inc version 20.0, Chicago, IL, USA).

## 3. Results

Ninety-four women with a mean age of 30.71 ± 2.61 years and a mean BMI of 23.9 ± 2.35 kg/m^2^ undergoing ART cycles were enrolled. Fifty-three was recruited from Federico II University, twenty-nine from Aarhus University, and twelve from the San Raffaele Institution. Hardy–Weinberg equilibrium was verified and the allele frequencies did not differ between the study group and the general population (Table 1). Baseline FSH serum levels were 6.73 ± 1.98 IU/L. All patients underwent r-HFSH 150 IU daily, according to the study protocol and the mean total r-hFSH dosage prescribed was 1725.33 ± 520.15 IU. Stimulation had an average duration of 11.24 ± 1.69 days (Table 2). Only two cycles (2.4%) were interrupted due to risk of OHSS, whereas no cycles were interrupted for non-response to OS. After OS, mean E2 serum levels were 1655.43 ± 895.59 pg/mL, and women underwent IVF in 28.7% of cases (27 women) and intracytoplasmatic sperm injection in 71.2% of cases (67 women) (Table 2). Forty pregnancies per cycle (42.5%) were obtained according to β-hCG measurement and 32 (34.04%) of these were diagnosed by ultrasound assessment (Table 2).

### 3.1. FSHR 307 (rs6165) and FSHR 680 (rs6166)

The total number of oocytes retrieved did not differ between carriers of FSHR rs6165 (*p* = 0.510) and carriers of FSHR rs6166 (*p* = 0.170). The ratio between total gonadotropin consumption and number of oocytes retrieved differed almost significantly among three genotypes (*p* = 0.050). As shown in Table 3 and Table 4, the following features did not differ between the two SNPs: total r-hFSH dosage used, the ratio between fertilized and inseminated oocytes, E2 levels on the day of hCG, cumulative dosage of r-hFSH, number of preovulatory follicles, mature oocytes retrieved (MII oocytes), number of fertilized oocytes, number of embryos transferred, implantation rate, pregnancy rate per cycle, pregnancy rate per transfer, clinical pregnancy rate for started cycle, clinical pregnancy rate per transfer.

### 3.2. FSHR-29 (rs1394205)

Treatment outcomes did not differ among models generated according to genotype frequencies (Table 5).

### 3.3. LHCGR 291 (rs12470652)

LHCGR heterozygous women had higher E2 levels on the day of hCG administration (*p* = 0.005) than wild-type carriers (Table 6). Similarly, the numbers of total retrieved oocytes (*p* = 0.035), MII (*p* = 0.002), inseminated oocytes (*p* = 0.001), fertilized oocytes (*p* = 0.001), and cryopreserved embryos (*p* = 0.001) were higher in heterozygous women than in wild-type carriers (Table 6). No significant differences were found among the other variables (Table 6).

### 3.4. LHCGR Intronic (rs4073366), LHCGR 312 (rs2293275), FSHB 2623 (rs6169), and v-LH (rs1800447)

The parameters evaluated did not differ significantly among LHCGR rs4073366, LHCGR rs2293275, FSHB rs10835638, and v-LH rs1800447 carriers (Supplemental Tables S1–S4).

### 3.5. Multivariate Analysis

In multivariate analysis, the copresence of allele G of FSHR-29 rs1394205 and allele C of LHCGR 291 rs12470652 was related to an increased ratio between cumulative r-hFSH dose and total number of oocytes retrieved (5.47, CI 95%: 3.13–7.81, *p* < 0.001). Furthermore, the copresence of allele G of both FSHR-29 rs1394205 and FSHR rs6166 and allele C LHCGR 291 rs12470652 was related to an increased ratio between cumulative FSH dose and total number of oocytes retrieved (5.44, CI: 3.18–7.71, *p* < 0.001).

## 4. Discussion

Our data support the concept that ovarian response to exogenous gonadotropin could be influenced by specific genetic traits. In detail, we observed that two common FSHR (FSHR rs6165 and rs6166) SNPs and one LHCGR (rs12470652) SNP might affect OS outcomes in terms of number of oocytes retrieved, cumulative r-hFSH dosage, and oocyte ratio. Furthermore, our multivariate analysis suggests that the interaction of specific genetic traits could also influence ovarian sensitivity to exogenous gonadotropin. To our knowledge, this is first time that LHCGR 291 (rs12470652) has been implicated in response to OS.

In contrast to Ackrekar et al. [9], we did not find any association between FSHR-29 polymorphism and OS response. This discordance might be due to the differences in study design, inclusion criteria, and protocols between the two studies. In fact, we conducted a prospective analysis adopting very strict inclusion criteria. In addition, we used a standardized OS protocol in which recombinant gonadotropin was used at a fixed starting dose.

Our finding that GG carriers are resistant to both endogenous and exogenous FSH is consistent with previous studies [2,6,7,8]. This resistance seems to be related to specific molecular characteristics. In fact, in vitro studies showed that women with a GG haplotype (rs6166) genotype have reduced sensitivity compared with the AA haplotype [25,26]. Unlike our previous studies [13,27], we did not detect an association between OS and the LH β polymorphism rs1800447. This incongruity could reflect the absence of homozygous carriers of this polymorphic trait in the present study and the reduced number of women recruited, namely, 94 patients versus 200 cases reported in 2013 [13]. It could also reflect the different designs of the two studies.

Our study has several strengths. Firstly, it was a multicenter prospective analysis conducted using detailed inclusion/exclusion criteria. Indeed, we enrolled only patients with an adequate ovarian reserve using a fixed starting daily dose who did not require “coasting” for hyper-response or multiple dosage adjustments during OS. We adopted these inclusion criteria to avoid any misinterpretation of data. Indeed, some authors demonstrated that elevated FSH dosage, as well as dose adjustment during OS, could mitigate the effect of genotype on ART [28]. Furthermore, we also carried out a multivariate analysis to establish whether interactions among different polymorphisms could influence ovarian response. Most previous studies focused on a single polymorphism [8,9,28,29], and only the most recent studies considered no more than two polymorphisms together [16,19]. Furthermore, ours is the first study to evaluate the LHCGR intronic (rs4073366) and FSHB 2623 (rs10835638) polymorphisms in ART.

The most important limitation of our investigation resides in the relatively small number of women enrolled. In addition, we were not able to follow patients up until childbirth, although we do provide data on the ongoing pregnancy rate. Like other studies, we did not find a significant association between gonadotropins and their polymorphisms and pregnancy outcomes [13,29,30]. However, this does not necessarily mean that gonadotropins did not affect OS. In fact, we believe that the number of oocytes retrieved and the amount of gonadotropin consumed during OS are more appropriate measures than ART births of the influence of gonadotropins and their receptor genetic variants in an in vitro fertilization setting. Indeed, ART births are influenced by such other factors as embryo quality and maternal age and factors that occur during pregnancy, namely, intrauterine growth restriction, that are poorly related to genetic variants affecting gonadotropins and their receptors. Furthermore, we enrolled women who carried out a long analogue protocol and consequently we cannot provide data about antagonist protocols that are currently widely adopted. Finally, another limitation is that we limited our analysis to young, good prognosis patients, thus our data could not be generalized to advanced age women that often require medically assisted reproduction.

## 5. Conclusions

In conclusion, our analysis supports the concept that distinct polymorphisms could modulate the response to OS. Moreover, we demonstrate that simultaneous analysis of multiple polymorphisms provides useful information about the response to controlled ovarian response. Our data need to be confirmed by further investigations, especially for LHCGR intronic (s4073366) and FSHB 2623 (rs10835638) polymorphisms. Despite our findings, more robust studies are required to establish the clinical utility of genotype analysis before ovarian stimulation.

## Figures and Tables

**Table 1:** Baseline characteristics of the population studied (*n* = 94).

Basal Characteristics	Values
Age (years)	30.71 ± 2.61
BMI (kg/m^2^)	22.94 ± 2.35
AMH (ng/mL)	2.70 ± 1.76
Antral follicle count	12.36 ± 3.63
Basal FSH (IU/L)	6.73 ± 1.98
Basal estradiol (pg/mL)	80.65 ± 101.16

**Table 2:** Treatment outcomes (*n* = 94).

Treatment Outcomes *	Values
Total FSH doses (IU)	1725.33 ± 520.15
Days of stimulation	11.24 ± 1.69
Estradiol at the day of hCG (pg/mL)	1655.43 ± 895.59
Follicles > 10 mm	11.04 ± 4.41
Follicles > 16 mm	7.72 ± 3.15
Oocyte number	9.51 ± 3.82
Mature oocyte number	7.78 ± 3.39
Oocytes inseminated	5.35 ± 3.50
Oocytes fertilized	3.61 ± 2.55
Oocytes cryopreserved	0.35 ± 1.36
Embryos cryopreserved	6.73 ± 1.98
Embryos transferred	1.65 ± 0.80
Cycles canceled for hyper-response	2 (2.1%)
OHSS	1 (1.1%)
Implantation rate	45.7%
Pregnancy rate (β-hCG) per cycle	42.5%
Ongoing pregnancy rate per cycle	34.04%
Pregnancy rate (β-hCG) per embryos transferred	30.5%
Ongoing pregnancy rate per embryos transferred	25.3%
Miscarriage rate per cycle	9.4%

* Continuous data are expressed as mean ± standard deviation; categorical data as percentage.

**Table 3:** Treatment outcomes in patients stratified according to the FSHR 307 (rs6165) polymorphism.

	Homozygous A/A (*n* = 24)	Heterozygous A/G (*n* = 50)	Homozygous G/G (*n* = 20)	*p*-Value
Total FSH doses (IU)	1781.23 ± 568.45	1730.04 ± 550.19	1647.17 ± 383.58	*0.536*
FSH/oocytes	243.42 ± 97.60	338.52 ± 251.80	252.60 ± 166.33	***0.050***
Days of stimulation	11.13 ± 1.68	11.35 ± 1.82	11.10 ± 1.41	*0.769*
Endometrial thickness (mm)	9.70 ± 1.15	10.38 ± 2.00	10.28 ± 2.09	*0.547*
Estradiol on the day of hCG (pg/mL)	1555.24 ± 663.85	1607.54 ± 906.21	1859.42 ± 1092.75	*0.513*
Follicles ≥ 16 mm on the day of hCG	7.63 ± 2.72	7.73 ± 3.26	7.80 ± 3.50	*0.983*
Oocyte number	9.58 ± 3.32	9.24 ± 3.57	10.10 ± 4.98	*0.685*
Mature oocyte number	8.13 ± 2.72	7.50 ± 3.71	8.06 ± 3.47	*0.643*
Oocytes inseminated	6.08 ± 3.26	5.18 ± 3.60	4.90 ± 3.55	*0.346*
Oocytes fertilized	3.92 ± 2.53	3.60 ± 2.66	3.25 ± 2.38	*0.537*
Oocytes cryopreserved	0.21 ± 1.02	0.36 ± 1.44	0.50 ± 1.54	*0.802*
Embryos cryopreserved	0.96 ± 1.81	1.16 ± 2.05	0.65 ± 1.31	*0.534*
Embryos transferred	1.63 ± 0.77	1.56 ± 0.79	1.90 ± 0.85	*0.236*
Implantation rate	10/39	23/77	10/38	*0.795*
Pregnancy rate per embryos transferred	12/39	24/77	11/38	*0.867*
Ongoing pregnancy rate per embryos transferred	9/39	21/77	9/38	*0.792*
Pregnancy rate per cycle	12/24	24/50	11/20	*0.930*
Ongoing pregnancy rate per cycle	9/24	21/50	9/20	*0.863*

Bold indicates statistical significance.

**Table 4:** Treatment outcomes in patients stratified according to the FSHR 680 (rs6166) polymorphism.

	Homozygous A/A (*n* = 24)	Heterozygous A/G (*n* = 49)	Homozygous G/G (*n* = 21)	*p*-Value
Total FSH doses (IU)	1809.76 ± 563.38	1725.25 ± 554.53	1633.02 ± 379.45	*0.698*
FSH/oocytes	248.80 ± 96.34	333.44 ± 250.88	252.60 ± 166.33	***0.049***
Days of stimulation	11.42 ± 1.72	11.23 ± 1.81	11.50 ± 1.40	*0.804*
Endometrial thickness (mm)	9.70 ± 1.15	10.38 ± 2.00	10.28 ± 2.09	*0.547*
Estradiol on the day of hCG (pg/mL)	1624.09 ± 722.40	1568.46 ± 880.90	1859.42 ± 1092.75	*0.514*
Follicles ≥ 16 mm on the day of hCG	7.58 ± 2.70	7.71 ± 3.28	7.90 ± 3.45	*0.944*
Oocyte number	9.67 ± 3.33	9.20 ± 3.59	10.05 ± 4.86	*0.697*
Mature oocyte number	8.22 ± 2.78	7.45 ± 3.67	8.06 ± 3.47	*0.725*
Oocytes inseminated	6.21 ± 3.35	5.18 ± 3.55	4.76 ± 3.52	*0.476*
Oocytes fertilized	4.04 ± 2.56	3.57 ± 2.65	3.19 ± 2.38	*0.694*
Oocytes cryopreserved	0.21 ± 1.02	0.37 ± 1.45	0.48 ± 1.50	*0.779*
Embryos cryopreserved	1.00 ± 1.82	1.16 ± 2.06	0.62 ± 1.28	*0.581*
Embryos transferred	1.63 ± 0.77	1.55 ± 0.79	1.90 ± 0.83	*0.273*
Implantation rate	10/37	21/77	12/40	*0.844*
Pregnancy rate per embryos transferred	12/37	22/77	13/40	*0.839*
Ongoing pregnancy rate per embryos transferred	9/37	19/77	11/40	*0.848*
Pregnancy rate per cycle	12/23	23/50	13/21	*0.812*
Ongoing pregnancy rate per cycle	9/23	19/50	11/21	*0.867*

Bold indicates statistical significance.

**Table 5:** Treatment outcomes in patients stratified according to the FSHR-29 (rs1394205).

	Homozygous G/G (*n* = 54)	Heterozygous G/A (*n* = 31)	Homozygous A/A (*n* = 8)	*p*-Value
Total FSH doses (IU)	1730.04 ± 554.55	1745.19 ± 476.55	1601.71 ± 487.46	*0.804*
FSH/oocytes	322.78 ± 239.49	229.73 ± 109.79	312.83 ± 161.03	*0.186*
Days of stimulation	11.20 ± 1.52	11.39 ± 1.87	11.50 ± 1.40	*0.733*
Endometrial thickness (mm)	10.29 ± 2.00	9.91 ± 1.55	10.47 ± 1.36	*0.776*
Estradiol on the day of hCG (pg/mL)	1518.52 ± 742.11	1832.04 ± 1184.50	2039.14 ± 706.30	*0.197*
Follicles ≥ 16 mm on the day of hCG	8.07 ± 3.23	6.90 ± 2.83	8.50 ± 3.50	*0.197*
Oocyte number	9.60 ± 3.90	9.58 ± 3.84	8.63 ± 3.54	*0.794*
Mature oocyte number	7.67 ± 3.65	8.46 ± 2.90	6.50 ± 2.83	*0.344*
Oocytes inseminated	5.22 ± 3.49	5.90 ± 3.67	4.13 ± 2.70	*0.404*
Oocytes fertilized	3.47 ± 2.67	3.97 ± 2.60	3.13 ± 1.36	*0.595*
Oocytes cryopreserved	0.38 ± 1.38	0.19 ± 1.08	0.75 ± 2.12	*0.572*
Embryos cryopreserved	1.05 ± 2.03	1.03 ± 1.72	0.50 ± 0.76	*0.730*
Embryos transferred	1.62 ± 0.85	1.61 ± 0.76	2.00 ± 0.53	*0.435*
Implantation rate	18/88	21/50	4/16	*0.934*
Pregnancy rate per embryos transferred	22/88	21/50	4/16	*0.754*
Ongoing pregnancy rate per embryos transferred	16/88	19/50	4/16	*0.770*
Pregnancy rate per cycle	22/55	21/31	4/8	*0.879*
Ongoing pregnancy rate per cycle	16/55	19/31	4/8	*0.435*

**Table 6:** Treatment outcomes in patients stratified according to the LHCGR 291 (rs12470652) polymorphism.

	T/T (*n* = 87)	C/T (*n* = 7)	*p*-Value
Total FSH doses (IU)	1736.38 ± 534.53	1568.75 ± 196.17	*0.449*
FSH/oocytes	305.86 ± 208.66	147.65 ± 46.77	*0.069*
Days of stimulation	11.21 ± 1.70	11.57 ± 1.62	*0.588*
Endometrial thickness (mm)	9.97 ± 1.49	11.04 ± 2.76	*0.146*
Estradiol on the day of hCG (pg/mL)	1580.60 ± 860.03	2733.00 ± 747.23	***0.005***
Follicles ≥ 16 mm on the day of hCG	7.80 ± 3.16	6.71 ± 2.98	*0.382*
Oocyte number	9.28 ± 3.81	12.43 ± 2.82	***0.035***
Mature oocyte number	7.45 ± 3.21	11.43 ± 3.41	***0.002***
Oocytes inseminated	4.92 ± 3.20	10.71 ± 2.56	***0.001***
Oocytes fertilized	3.24 ± 2.16	8.14 ± 2.91	***0.001***
Oocytes cryopreserved	0.38 ± 1.41	0.00 ± 0.00	*0.480*
Embryos cryopreserved	0.75 ± 1.47	4.14 ± 3.08	***0.001***
Embryos transferred	1.68 ± 0.81	1.29 ± 0.49	*0.213*
Implantation rate	41/145	2/9	*0.992*
Pregnancy rate per embryos transferred	45/145	4/9	*0.639*
Ongoing pregnancy rate per embryos transferred	37/145	2/9	*0.861*
Pregnancy rate per cycle	45/87	4/7	*0.907*
Ongoing pregnancy rate per cycle	37/87	2/7	*0.747*

Bold indicates statistical significance.

## Data Availability

Data are unavailable due to privacy restrictions.

## Supplementary Materials

The following supporting information can be downloaded at: https://www.mdpi.com/article/10.3390/genes14061269/s1, Table S1: Treatment outcomes. Patients stratified according to the rs LHCGR intronic polymorphism (rs4073366); Table S2: Treatment outcomes in patients stratified according to the LHCGR 312 (rs2293275) polymorphism; Table S3: Treatment outcomes in patients stratified according to the rs FSHB 2623 (rs6169) polymorphism; Table S4: Treatment outcomes in patients stratified according to the LHB (rs1800447) polymorphism.
