# Supplementary material for: Genetic Variants of Gonadotropins and Their Receptors Could Influence Controlled Ovarian Stimulation: IVF Data from a Prospective Multicenter Study
Source: Genes (Basel). 2023 Jun 15;14(6):1269. doi: 10.3390/genes14061269 (PMC10298688; doi:10.3390/genes14061269)
Supplement: Supplementary file 1 [file genes-14-01269-s001.zip › Supplemental Table S1_JAG.pdf]

Supplemental Table S1: Treatment outcomes. Patients stratified according to the rs LHCGR intronic polymorphism (rs4073366).

	**Homozygous C/C**	**Heterozygous C/G**	**Homozygous G/G**	***p-value***
Total FSH doses (IU)	1751.42±565.69	1661.43±441.10	1800.00±150.00	*0.732*
FSH/oocytes	300.74±215.01	286.65±194.49	214.29±90.91	*0.826*
Days of stimulation	11.18±1.63	11.29±1.88	12.00±1.00	*0.705*
Endometrial thickness (mm)	9.93±1.54	10.46±2.12	11.05±2.76	*0.525*
Estradiol at the day of hCG (pg/mL)	1555.90±765.64	1876.68±1112.63	1200.00±1000.00	*0.303*
Follicles ≥ 16mm at the of day of HCG	7.82±2.93	7.36±3.71	9.00±1.73	*0.632*
Oocyte number	9.56±4.10	9.38±3.41	9.67±2.31	*0.975*
Mature oocyte number	7.74±3.40	7.78±3.49	9.00±2.83	*0.878*
Oocytes inseminated	5.24±3.49	5.41±3.56	7.00±4.00	*0.696*
Oocytes fertilized	3.60±2.61	3.38±2.14	6.00±4.58	*0.241*
Oocytes cryopreserved	0.35±1.37	0.38±1.42	0.00±0.00	*0.901*
Embryos cryopreserved	0.95±1.81	1.10±2.01	1.00±1.73	*0.937*
Embryos transferred	1.69±0.84	1.59±0.73	1.33±0.58	*0.662*
Implantation rate	32/104	10/46	1/4	*0.348*
Pregnancy rate per embryo transferred	33/104	13/46	1/4	*0.816*
Ongoing pregnancy rate per embryo transferred	29/104	9/46	1/4	*0.381*
Pregnancy rate per cycle	33/62	13/29	1/3	*0.563*
Ongoing pregnancy rate per cycle	29/104	9/29	1/3	*0.656*
