# Supplementary material for: Genetic Variants of Gonadotropins and Their Receptors Could Influence Controlled Ovarian Stimulation: IVF Data from a Prospective Multicenter Study
Source: Genes (Basel). 2023 Jun 15;14(6):1269. doi: 10.3390/genes14061269 (PMC10298688; doi:10.3390/genes14061269)
Supplement: Supplementary file 1 [file genes-14-01269-s001.zip › Supplemental Table S2_JAG.pdf]

Supplemental Table S2: Treatment outcomes in patients stratified according to the LHCGR 312 (rs2293275) polymorphism.

	**Homozygous T/T**	**Heterozygous T/C**	**Homozygous C/C**	***p-value***
Total FSH doses (IU)	1807.00±667.15	1703.23±451.24	1713.54±533.65	*0.788*
FSH/oocytes	322.28±169.80	241.81±164.53	329.30±246.56	*0.195*
Days of stimulation	11.18±1.78	11.29±1.68	11.20±1.71	*0.960*
Endometrial thickness (mm)	10.08±2.73	10.08±1.46	10.32±1.49	*0.925*
Estradiol at the day of hCG (pg/mL)	1773.50±645.43	1731.31±1104.56	1523.77±748.41	*0.571*
Follicles ≥ 16mm at the day of hCG	7.12±3.43	7.24±2.75	8.57±3.35	*0.127*
Oocyte number	8.47±2.98	9.86±4.11	9.60±3.84	*0.449*
Mature oocyte number	7.29±3.60	8.72±3.29	7.15±3.27	*0.136*
Oocytes inseminated	5.53±3.52	5.93±3.87	4.57±2.91	*0.233*
Oocytes fertilized	3.29±2.52	4.05±3.10	3.23±1.68	*0.324*
Oocytes cryopreserved	0.00±0.00	0.14±0.93	0.77±1.93	*0.063*
Embryos cryopreserved	1.24±2.11	1.10±2.02	0.77±1.50	*0.636*
Embryos transferred	1.41±0.87	1.55±0.67	1.89±0.87	*0.071*
Implantation rate	4/23	16/65	23/66	*0.192*
Pregnancy rate per embryo transferred	5/24	19/65	23/66	*0.311*
Ongoing pregnancy rate per embryo transferred	3/24	15/65	21/66	*0.118*
Pregnancy rate per cycle	5/17	19/42	23/35	*0.408*
Ongoing pregnancy rate per cycle	3/17	15/42	21/35	*0.292*
