# Supplementary material for: Genetic Variants of Gonadotropins and Their Receptors Could Influence Controlled Ovarian Stimulation: IVF Data from a Prospective Multicenter Study
Source: Genes (Basel). 2023 Jun 15;14(6):1269. doi: 10.3390/genes14061269 (PMC10298688; doi:10.3390/genes14061269)
Supplement: Supplementary file 1 [file genes-14-01269-s001.zip › Supplemental Table S3_JAG.pdf]

**Supplemental Table S3:** Treatment outcomes in patients stratified according to the rs FSHB 2623 (rs6169) polymorphism.

| | **Homozygous T/T** | **Heterozygous T/C** | **Homozygous C/C** | ***p-value*** |
|---|---|---|---|---|
| Total FSH doses (IU) | 1777.94±510.79 | 1677.91±513.57 | 1782.50±550.64 | *0.648* |
| FSH/oocytes | 300.14±310.68 | 287.17±175.63 | 301.22±179.14 | *0.958* |
| Days of stimulation | 10.88±1.41 | 11.12±1.45 | 11.69±2.03 | *0.240* |
| Endometrial thickness (mm) | 10.30±0.91 | 9.97±1.77 | 10.31±2.06 | *0.844* |
| Estradiol on the day of hCG (pg/mL) | 1085.42±679.11 | 1679.86±789.85 | 1929.95±895.59 | *0.300* |
| Follicles ≥ 16mm on the day of hCG | 8.29±3.51 | 7.72±2.96 | 7.35±3.32 | *0.633* |
| Oocyte number | 10.24±4.26 | 9.16±3.58 | 9.73±4.04 | *0.572* |
| Mature oocyte number | 8.53±3.52 | 7.55±3.23 | 7.73±3.63 | *0.629* |
| Oocytes inseminated | 5.88±4.06 | 4.80±3.12 | 6.08±3.75 | *0.254* |
| Oocytes fertilized | 4.24±3.03 | 3.27±2.23 | 3.85±2.81 | *0.350* |
| Oocytes cryopreserved | 0.29±1.21 | 0.22±1.08 | 0.65±1.85 | *0.405* |
| Embryos cryopreserved | 1.53±2.24 | 0.67±1.27 | 1.31±2.40 | *0.152* |
| Embryos transferred | 1.71±0.92 | 1.67±0.82 | 1.58±0.70 | *0.854* |
| Implantation rate | 12/29 | 19/84 | 12/41 | *0.922* |
| Pregnancy rate per embryo transferred | 12/29 | 21/84 | 14/41 | *0.933* |
| Ongoing pregnancy rate per embryo transferred | 10/29 | 17/84 | 12/41 | *0.821* |
| Pregnancy rate per cycle | 12/17 | 21/51 | 14/26 | *0.436* |
| Ongoing pregnancy rate per cycle | 10/17 | 17/51 | 12/26 | *0.617* |
