# Supplementary material for: Genetic Variants of Gonadotropins and Their Receptors Could Influence Controlled Ovarian Stimulation: IVF Data from a Prospective Multicenter Study
Source: Genes (Basel). 2023 Jun 15;14(6):1269. doi: 10.3390/genes14061269 (PMC10298688; doi:10.3390/genes14061269)
Supplement: Supplementary file 1 [file genes-14-01269-s001.zip › Supplemental Table S4_JAG.pdf]

Supplemental Table S4: Treatment outcomes in patients stratified according to the LHB (rs1800447) polymorphism.

	**T/T**	**T/C**	***p-value***
Total FSH doses (IU)	1766.44±557.28	1579.37±329.68	*0.157*
FSH/oocytes	301.90±214.61	266.76±171.73	*0.526*
Days of stimulation	11.37±1.78	10.75±1.21	*0.147*
Endometrial thickness (mm)	10.24±1.43	9.84±2.75	*0.563*
Estradiol on the day of hCG (pg/mL)	1586.26±860.00	1941.33±1011.35	*0.170*
Follicles ≥ 16mm on the of hCG	7.47±3.02	8.65±3.51	*0.137*
Oocyte number	9.41±3.61	9.90±4.62	*0.610*
Mature oocyte number	7.69±3.21	8.11±4.07	*0.646*
Oocytes inseminated	5.30±3.17	5.55±4.59	*0.776*
Oocytes fertilized	3.47±2.33	4.10±3.27	*0.333*
Oocytes cryopreserved	0.22±1.06	0.85±2.08	*0.064*
Embryos cryopreserved	0.81±1.59	1.70±2.51	*0.056*
Embryos transferred	1.69±0.86	1.50±0.51	*0.350*
Implantation rate	35/126	8/28	*0.822*
Pregnancy rate per embryo transferred	36/126	11/28	*0.375*
Ongoing pregnancy rate per embryo transferred	32/126	7/28	*0.844*
Pregnancy rate per cycle	36/75	11/19	*0.607*
Ongoing pregnancy rate per cycle	32/75	7/19	*0.842*
